# Species Interaction and Selective Carbon Addition During Antibiotic Exposure Enhances Bacterial Survival

**DOI:** 10.3389/fmicb.2019.02730

**Published:** 2019-11-29

**Authors:** Lindsay M. D. Jackson, Otini Kroukamp, William C. Yeung, Evan Ronan, Steven N. Liss, Gideon M. Wolfaardt

**Affiliations:** ^1^Department of Chemistry and Biology, Ryerson University, Toronto, ON, Canada; ^2^Department of Microbiology, Stellenbosch University, Stellenbosch, South Africa

**Keywords:** biofilm, mixed-species, environmental changes, carbon, *Pseudomonas aeruginosa*, *Stenotrophomonas maltophilia*

## Abstract

Biofilms are multifaceted and robust microbiological systems that enable microorganisms to withstand a multitude of environmental stresses and expand their habitat range. We have shown previously that nutritional status alters antibiotic susceptibility in a mixed-species biofilm. To further elucidate the effects of nutrient addition on inter-species dynamics and whole-biofilm susceptibility to high-dose streptomycin exposures, a CO_2_ Evolution Measurement System was used to monitor the metabolic activity of early steady state pure-culture and mixed-species biofilms containing *Pseudomonas aeruginosa* and *Stenotrophomonas maltophilia*, with and without added carbon. Carbon supplementation was needed for biofilm recovery from high-dose streptomycin exposures when *P. aeruginosa* was either the dominant community member in a mixed-species biofilm (containing predominantly *P. aeruginosa* and *S. maltophilia*) or as a pure culture. By contrast, *S. maltophilia* biofilms could recover from high-dose streptomycin exposures without the need for carbon addition during antibiotic exposure. Metagenomic analysis revealed that even when inocula were dominated by *Pseudomonas*, the relative abundance of *Stenotrophomonas* increased upon biofilm development to ultimately become the dominant species post-streptomycin exposure. The combined metabolic and metagenomic results demonstrated the relevance of inter-species influence on survival and that nutritional status has a strong influence on the survival of *P. aeruginosa* dominated biofilms.

## Introduction

Biofilms are multifaceted and robust microbiological systems that enable microorganisms to withstand a multitude of environmental stressors (Marchand et al., 2012; Tan et al., 2017). Natural biofilms typically consist of multispecies communities that cooperate (Davey and O’Toole, 2000) to enhance their success in survival (de Carvalho, 2018). For example, bacterial strains isolated from the surface of marine algae demonstrated enhanced biofilm growth and resistance to antimicrobial agents when compared to any of the species grown in pure culture (Burmølle et al., 2006). Another examples of beneficial interspecies interactions come from a study that demonstrated the opportunistic pathogen *S. maltophilia* aided in the survival of susceptible strains of *Pseudomonas aeruginosa* and *Serratia marcescens* in the presence of beta-lactam antibiotics (Kataoka, 2003), and the study by Ronan et al. (2013), which show that an *Arthrobacter* species greatly extends the desiccation tolerance of *Pseudomonas aeruginosa* after deposition on dry surfaces. Although biofilms are highly beneficial to geochemical cycling and other environmental processes necessary for human survival (Paerl and Pinckney, 1996) and ecosystem services, they can have grave socioeconomic impacts when controlled environments are necessary, such as in industrial or clinical settings (Marchand et al., 2012; de Carvalho, 2018). In clinical settings, biofilms are difficult to eradicate with conventional antibiotic treatment regimens (Costerton et al., 1999; Mah and O’Toole, 2001; Nithya et al., 2010), and billions of dollars in health care costs are incurred each year due to the development of chronic infections (Bryers, 2008; Beceiro et al., 2013).

The success of biofilms in resisting antibiotic treatment can be attributed in large part to biofilms having resistance mechanisms common to all bacteria as well as biofilm-specific resistance mechanisms. Bacterial resistance mechanisms include decreased outer-membrane permeability, efflux-pumps, expression and accumulation of antibiotic modifying and degrading enzymes, as well as alterations of antibiotic targets (Brooun et al., 2000; O’Toole et al., 2000; Bryers, 2008). Well-established biofilm specific resistance mechanisms include increased heterogeneity, poor antibiotic penetration, slower growth, nutrient limitation, adaptive stress responses, and the presence of persister cell (Stewart, 2002; Lewis, 2007). More recently, studies have also demonstrated how nutrient addition can either enhance or diminish antibiotic effects depending on the physiological states of the bacteria and the molecules present during exposure. For example, carbohydrate addition can act synergistically with antibiotics to enhance persister cells susceptibility (Allison et al., 2011), while the production of indole can upregulate oxidative stress genes and efflux pumps that increase antibiotic resistance in *Escherichia coli* cultures (Lee et al., 2010). Furthermore, biofilm studies have demonstrated that carbon addition can antagonize antibiotic effects on multispecies biofilms (Jackson et al., 2015), and phenazine addition to *P. aeruginosa* (PA14) biofilms can antagonize the effects of several classes of antibiotics (Schiessl et al., 2019).

**Figure 1:**
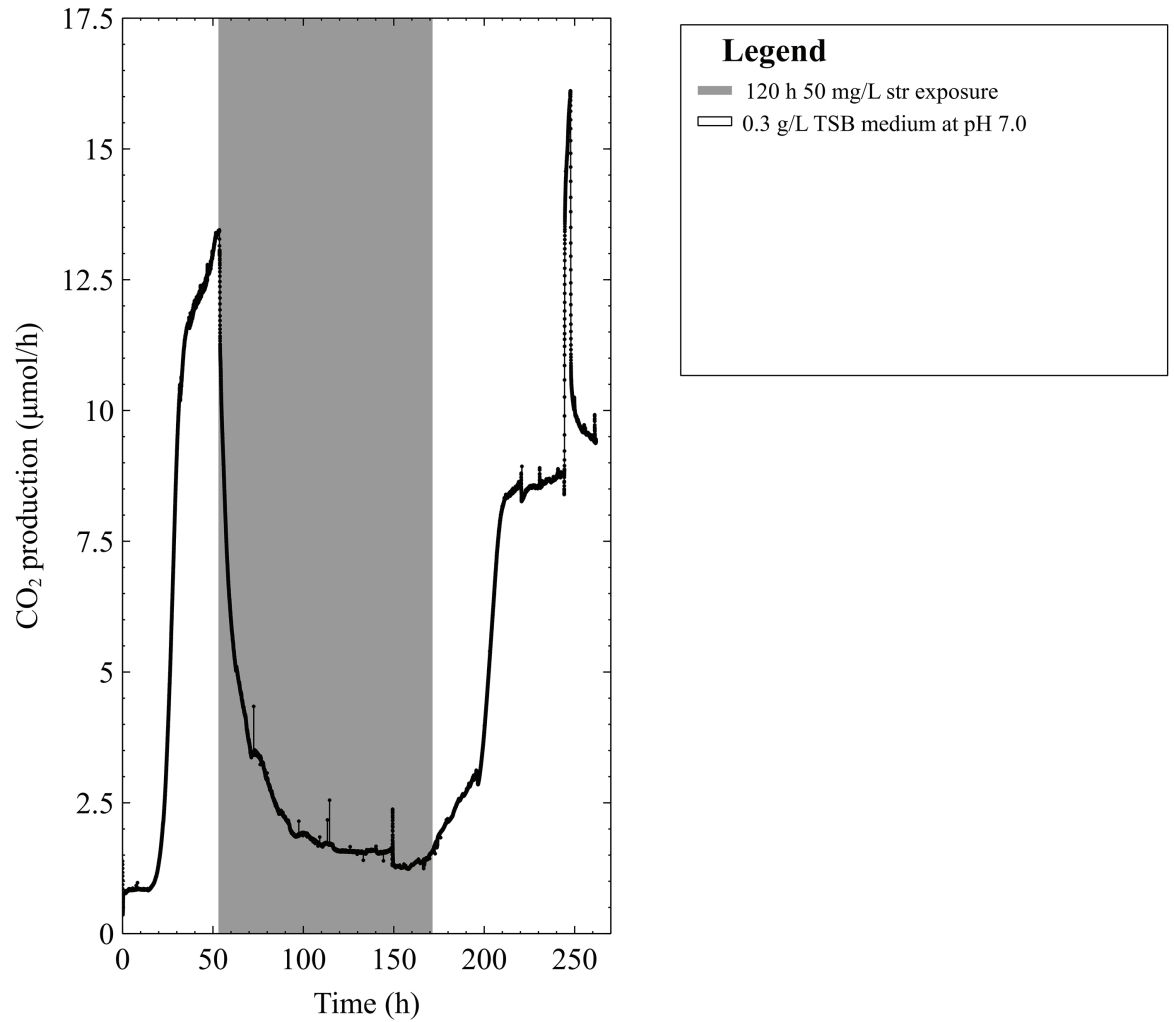
The CO_2_ production profile (μmol/h) of early steady state mixed-species biofilms grown in 0.3 g/L TSB medium exposed to 50 mg/L of streptomycin (str) for 120 h in 0.3 g/L TSB.

Previous work by (Jackson et al., 2015) demonstrated that multispecies biofilms (containing *P. aeruginosa* and *S. maltophilia*) could recover from high dose streptomycin (4,000 mg/L: more than 1,000 times higher than reported MICs) upon the addition of a carbon source that could be readily metabolized. Furthermore, when readily metabolized carbon sources were present during antibiotic exposure, characteristic increases in metabolism were observed and were predictive of biofilm recovery. In addition, literature states that *P. aeruginosa* and *S. maltophilia* employed different routes for glycolysis (Kanehisa and Goto, 2000b; Kanehisa et al., 2016) (the early stages of energy metabolism). Given these three observations, it was hypothesized that individual microbial species (pure culture) biofilms will demonstrate differential susceptibility or resistance against streptomycin based on their ability to rapidly degrade a readily available carbon source in the growth medium. Given this hypothesis we predicted that if a pure culture biofilm is susceptible to streptomycin in one growth medium, the addition of a readily metabolized carbon source to the medium could confer resistance in the biofilm. Indeed, we saw that pure culture *P. aeruginosa* did not survive streptomycin exposure in the growth medium [diluted trypticase soy broth (TSB)] used previously; or with the addition of more glucose (the carbon source natively present in TSB). However, the pure culture *P. aeruginosa* biofilm did survive the streptomycin exposure upon pyruvate addition. Lower concentration streptomycin exposures (50 mg/L) were tested; the biofilm metabolism never fully ceased throughout a 5-day continuous exposure and metabolism began increasing before the antibiotics were removed (Figure 1). Therefore, we decided to continue with the high-dose streptomycin exposures previously tested.

Since previous work showed carbon addition alongside an increase in metabolism could antagonize streptomycin exposures in a mixed-species biofilm we wanted to uncover (1) how the members of the biofilm community might behave independently of each other, i.e., in pure cultures; (2) the species composition of the untreated multispecies biofilm at various stages of biofilm development; and (3) how the species composition of the biofilms correlated to recovery from the antibiotics. The two common nosocomial pathogens *Pseudomonas aeruginosa* and *Stenotrophomonas maltophilia*, which are implicated and have been co-isolated in a number of diseases such as cystic fibrosis, venous leg ulcers, and hospital acquired pneumonia (Stover et al., 2000; Goss et al., 2002; Dowd et al., 2008; Tseng et al., 2009; Araoka et al., 2010; Brooke, 2012), were used in this work.

In order to determine how carbon affected the response of these two species to streptomycin exposure, the behavior of mixed-species biofilms comprising *P. aeruginosa* PAO1 (predominantly) and *S. maltophilia* was compared with their perspective behaviors in pure culture. The mixed-species biofilm was discovered when we came across a culture of PAO1 that demonstrated different behaviors in metabolism compared to other pure cultures of PAO1. Our rationale for pursuing experiments with this “contaminated” culture was that it potentially provides a better proxy for clinical infections, which almost invariably contain more than one species. A CO_2_ evolution measurement system (CEMS) was used to track whole-biofilm metabolism and subsequent metabolic changes in response to antibiotic exposures with and without the addition of different carbon sources. Metagenomic analysis before, during, and after antibiotic exposure revealed that the community composition changed during biofilm development and in response to antibiotic exposure. We demonstrate here that biofilms can survive high-dose streptomycin exposures through active metabolism linked to carbon addition during exposure. Our research proposes that the nutritional environment and species composition of the biofilm at the time of exposure are important determinants of subsequent survival. This study further proposes that survival of an actively metabolizing member can protect other susceptible species within the biofilm.

## Materials and Methods

### Strains and Culture Conditions

The bacterial strains used for biofilm experiments included *P. aeruginosa* PAO1 (Fraud and Poole, 2011) and *S. maltophilia* 560 (DSM 24970). Pure cultures of *S. maltophilia* and *P. aeruginosa* were maintained at −80°C and inoculated directly into 3 g/L tryptic soy broth (TSB) medium for overnight growth at 37°C and subsequent biofilm inoculation.

In addition to pure culture biofilms, a mixed-species biofilm comprising mainly of *Pseudomonas* and *Stenotrophomonas* strains, as confirmed by metagenomic analysis, was used (Jackson et al., 2015). Other species detected at very low numbers in the community *via* metagenomic analysis included *Xanthomonas*, *Burkholderia*, and microvirus. These bacteria are common contaminants in DNA extraction kits (Salter et al., 2014), and their presence in the culture is less certain than those of the more dominant members (*Pseudomonas* and *Stenotrophomonas*). The mixed-species culture was kept in freezer stocks at −20°C in 3 g/L tryptic soy broth (TSB) (EMD Chemicals, Billerica, MA, USA).

### Biofilm Cultivation

Biofilms were cultivated aerobically at 25°C in the CEMS apparatus described below (Bester et al., 2010). The CEMS apparatus allows for the real-time monitoring of whole-biofilm metabolism (Kroukamp and Wolfaardt, 2009). The CEMS was chosen as a method of measuring the rapid changes in biofilm metabolism in response to antibiotic exposures and subsequent whole biofilm metabolic response following antibiotic exposures. All biofilms were inoculated into the CEMS from an overnight culture, initially with 30 min of no flow to facilitate attachment. In the CEMS, a continuous flow of growth medium was fed into the gas-permeable inner silicone tubing (inside diameter, 0.16 cm; outside diameter, 0.24 cm; length, 150 cm; VWR International, Mississauga, ON, Canada), which is encased in a gas impermeable Tygon tube. The CO_2_ produced by the biofilm crossed the silicone tube wall and was transported by CO_2_-free air (TOC grade < 0.5 ppm CO_2_, Linde, CA, USA) as sweeper gas and measured in real time with a CO_2_ analyzer (Li-Cor Biosciences, NE, USA; Bester et al., 2010). The 0.3 g/L TSB was continuously supplied to the biofilms at a flow rate of 15 ml/h (hydraulic retention time of 8 min). At this flow rate, planktonic cells were washed away faster than they could multiply within the CEMS apparatus, as the flow rate exceeds the planktonic specific growth rates by at least 10 times.

For our CEMS set up, the system was operated aerobically, and media were fed at a flow rate of 15 ml/h. Air was present in both the sweeper gas and media fed into the biofilm. The inner diameter of the silicone tubing used was 0.16 cm, and the biofilm thickness under these nutrient and flow conditions would average ~10 μm (Ronan et al., 2016). The CO_2_ produced in the experimental system was attributed to aerobic metabolism. To our knowledge, there were no alternative electron acceptors (apart from O_2_), so we do not expect significant anaerobic metabolism. *Pseudomonas* may ferment (pyruvate and arginine) under certain conditions, but it will lead to insignificant CO_2_ contributions since pyruvate fermentation cannot support growth, and our conditions are not conducive for arginine fermentation (Eschbach et al., 2004; Schreiber et al., 2006).

### Biofilm Antibiotic Exposures

It is commonly stated that biofilms can resist antibiotic concentrations up to 1,000x their planktonic MICs and that older biofilms are more resistant to antibiotics (Stewart, 2002). Furthermore, previous research has reported planktonic MICs of *S. maltophilia* strains to aminoglycosides (including streptomycin) in the thousands of mg/L. Thus, it was presumed that biofilms containing *S. maltophilia* might survive aminoglycoside concentrations in the thousands of mg/L (Zhang et al., 2000). The initial experimental run employed a mixed-species biofilm grown to steady-state metabolism (between 24 and 48 h after the initial growth period following exponential growth (Jackson et al., 2015) prior to being exposed to 50 mg/L streptomycin (inoculum planktonic MIC = 3.5 mg/ml) for 120 h. Since the biofilm rapidly recovered following exposure to the antibiotic (i.e. antibiotic withdrawn from the feed) at 50 mg/L, we tested what might happen if we exposed an older biofilm (5 days of growth) to a dose of antibiotic 1,000× the planktonic MIC. The mixed species biofilm exposed to 50 mg/L streptomycin began recovery prior to removal of the antibiotic medium and metabolic activity was observed to return to steady-state conditions upon resumption of antibiotic-free medium. For the older biofilm exposures, two separate biofilms that were grown to either 96 or 120 h and exposed to 4,000 mg/L were able to recover back to steady state levels within 72 h. Thus, the remainder of our experiments was high-dose streptomycin exposures performed on early steady state biofilms to monitor the early phases in which biofilms remain sensitive to high-dose streptomycin exposures.

Where duplicate and triplicate experiments were performed the graphs shown in the results section are showing CO_2_ outputs of single experimental runs, representative of what we have seen in replicates. Mixed species biofilms were grown to early steady state levels prior to exposure to 4,000 mg/L streptomycin for 4 h, in triplicate. Due to lack of early steady-state mixed species biofilm recovery following high-dose streptomycin exposures two experimental runs with carbon added during the antibiotic exposure were performed: the first run was with 2 mM pyruvate added, and the second run had 0.86 mM of glucose added to the antibiotic medium.

The added carbon aided in recovery of the multispecies biofilm, which then led us to explore the response of pure culture biofilms to the high-dose streptomycin exposures with and without added carbon. In duplicate for each strain, early steady-state pure-culture biofilms of *P. aeruginosa* PAO1 and *S. maltophilia* 560 were exposed to 4,000 mg/L streptomycin (added to the 0.3 g/L TSB medium) for 4 h. Next, in duplicate for each carbon source, pure-culture early steady-state PAO1 biofilms were exposed to 4,000 mg/L streptomycin in 0.3 g/L TSB medium supplemented with both pyruvate and glucose to a final concentration of 2 and 0.86 mM, respectively.

The use of streptomycin in this work was to demonstrate biofilm responses to stress under varying environmental conditions. It is well understood that (1) streptomycin is not used to treat chronic infections of either *P. aeruginosa* or *S. maltophilia* and (2) the levels of antibiotic used in this study greatly exceeded safe therapeutic levels in humans. The antibiotic medium was adjusted to pH 6.0 to keep consistent with previous work, since pH affects aminoglycoside susceptibility (Damper and Epstein, 1981; Xiong et al., 1996; Jackson et al., 2015). Streptomycin sulfate (Biobasic Inc. Markham, ON, Canada) was added directly to the various media, and the final pH was adjusted to pH 6.0–6.1 with 1 M HCl. The use of pyruvate in the addition of carbon experiments was because pyruvate is representative of the preferred carbon sources of *Pseudomonas* (Rojo, 2010), and because it is an end product of glycolysis and starting point of the Krebs cycle, where under aerobic conditions, it is converted into Acetyl-CoA. Under anaerobic condition, pyruvate can be converted to lactate with lactate dehydrogenase, which is found in nearly all living cells.

It is important to state here that these experiments are not high-throughput experiments where large numbers of replicates can be performed. However, this method of experimentation allows for real-time and automated observations of systems in flux, which removes the potential for errors arising due to experimental manipulation that can occur with microscopy and microtiter plate methods.

### Mixed-Species Whole-Biofilm and Biofilm Effluent Sample Preparation for DNA Extraction

Whole biofilms were sacrificed after 72 h for DNA extraction following the protocol described by Bester et al. (2010). Briefly, the entire bulk fluid (~2 ml) was drained from the CEMS into a waste container. Following removal of the bulk liquid, the remaining attached biofilm was removed by injecting 2 ml of a 0.1 M NaOH solution preheated to 60°C and collected into a 15 ml polyethylene tube. To ensure efficient biofilm collection and removal, another 2 ml of 0.1 M NaOH was injected into the biofilm growth chamber of the CEMS and kept in a 60°C incubator for 1 h prior to being flushed into the same 15 ml polyethylene tube containing the first wash of biofilm.

Biofilm effluent samples were collected prior to biofilm sampling for 18 h into sealed sterile flasks on ice. The biofilm fraction and biofilm effluent samples were centrifuged for 5 min at 12,000 ×*g*, and DNA extraction was performed on the cell pellets.

### Preparation of Genomic DNA for Full Genome Sequencing

Planktonic cultures from the mixed-species stock were grown in 3 g/L TSB medium and biofilms were grown in 0.3 g/L TSB medium. The genomic DNA of the planktonic cultures, whole-biofilm, and biofilm effluent collected from pre- and post-streptomycin exposed steady state biofilms were extracted using the MoBio UltraClean^®^ Microbial DNA Extraction Kit (MoBio Laboratories INC., Carlsbad, CA, USA) following the manufacturer’s protocol.

Early steady state biofilms in 0.3 g/L TSB media did not survive antibiotic exposures, thus older biofilms were used to capture pre-/post-exposure biofilm effluent for metagenomic analysis. Previous experimental data (unpublished work) found no culturable effluent cells post-antibiotic exposure. Thus, the only way to capture cells in the effluent post-antibiotic exposure was be to do it on an older biofilms in which you know the biofilm is capable of surviving and producing effluent cells.

### Metagenomic Analyses

Whole genome sequencing was performed by the Center for the Analysis of Genome Evolution and Function (CAGEF) at the University of Toronto, Toronto, Canada with a MiSeq Personal Sequencer v2. The unassembled data provided by CAGEF were analyzed by the mg-RAST server (v3.2), which provides annotation, analyses, and metadata on the metagenomes (Meyer et al., 2008).

## Results

We reported previously (Jackson et al., 2015) that early steady state multispecies biofilms, containing predominantly *P. aeruginosa* and *S. maltophilia*, survived high doses (4,000 mg/L) of streptomycin when a readily degradable (labile) carbon source was available. It was postulated that one or more strains in the multispecies culture were able to benefit from the presence of a readily degradable carbon source upon antibiotic addition, which aided in biofilm survival. To test our hypothesis that each species in the biofilm will demonstrate differential susceptibility to streptomycin, we investigated pure culture biofilms of *P. aeruginosa* and *S. maltophilia* to assess their ability to rapidly utilize a readily available carbon source in the growth medium.

**Figure 2:**
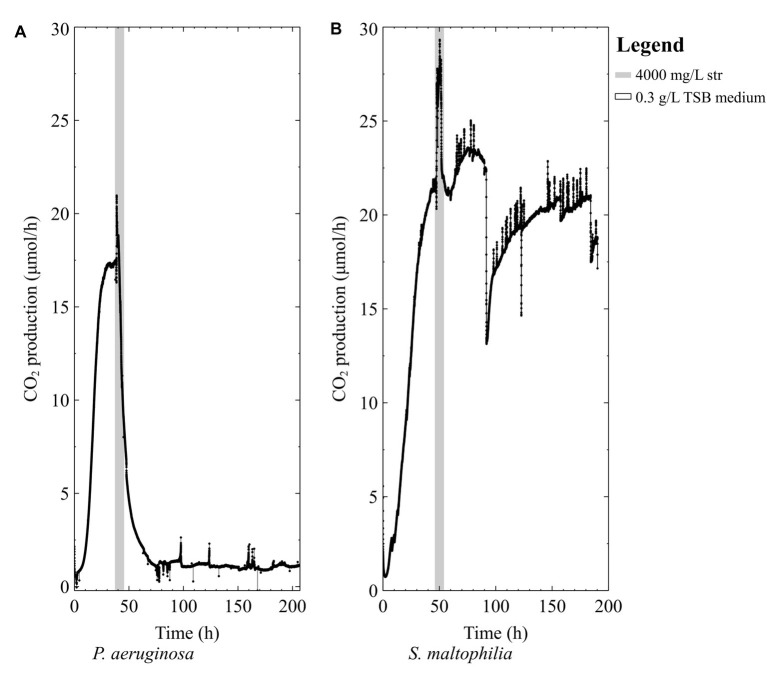
The CO_2_ production profile (μmol/h) of early steady state biofilms grown in 0.3 g/L TSB medium exposed to 4,000 mg/L of streptomycin (str) for 4 h in 0.3 g/L TSB **(A)** *P. aeruginosa* PAO1 and **(B)** *S. maltophilia* 560.

When pure-culture *P. aeruginosa* biofilms were grown in TSB medium, the biofilms were unable to survive the high-dose streptomycin exposures (Figure 2A). Conversely, when pure-culture *S. maltophilia* biofilms were grown in TSB medium and exposed to high-dose streptomycin, biofilms could recover from the antibiotic exposure (Figure 2B).

**Figure 3:**
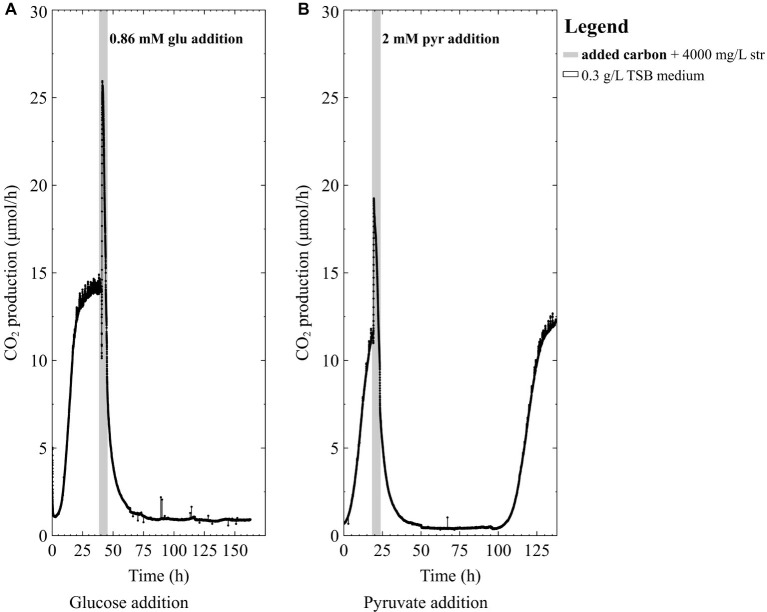
The CO_2_ production profile (μmol/h) of *P. aeruginosa* (PAO1) biofilms grown in 0.3 g/L TSB medium exposed to 4,000 mg/L streptomycin (str) with either **(A)** 0.86 mM glucose (glu) added for 4 h in 0.3 g/L TSB medium or **(B)** 2 mM pyruvate (pyr) added for 4 h in 0.3 g/L TSB medium.

Since pure-cultures of *P. aeruginosa* were unable to survive high doses of streptomycin in the TSB medium, we then tested the hypothesis that a nutritional boost in the form of a readily degradable carbon course could decrease biofilm susceptibility to high-dose streptomycin exposures. Early-steady state *P. aeruginosa* biofilms exposed to high doses of streptomycin did not recover when glucose was added as a nutritional boost (Figure 3A). On the other hand, the addition of 2 mM pyruvate to the antibiotic medium resulted in pure-culture *P. aeruginosa* biofilm recovery (Figure 3B), a number of days after exposure. Given the variations in survival of the pure culture biofilms, we set out to explore the effect of carbon addition during high-dose streptomycin exposures on mixed-species biofilms containing a higher proportion of *P. aeruginosa* (the more susceptible community member).

**Figure 4:**
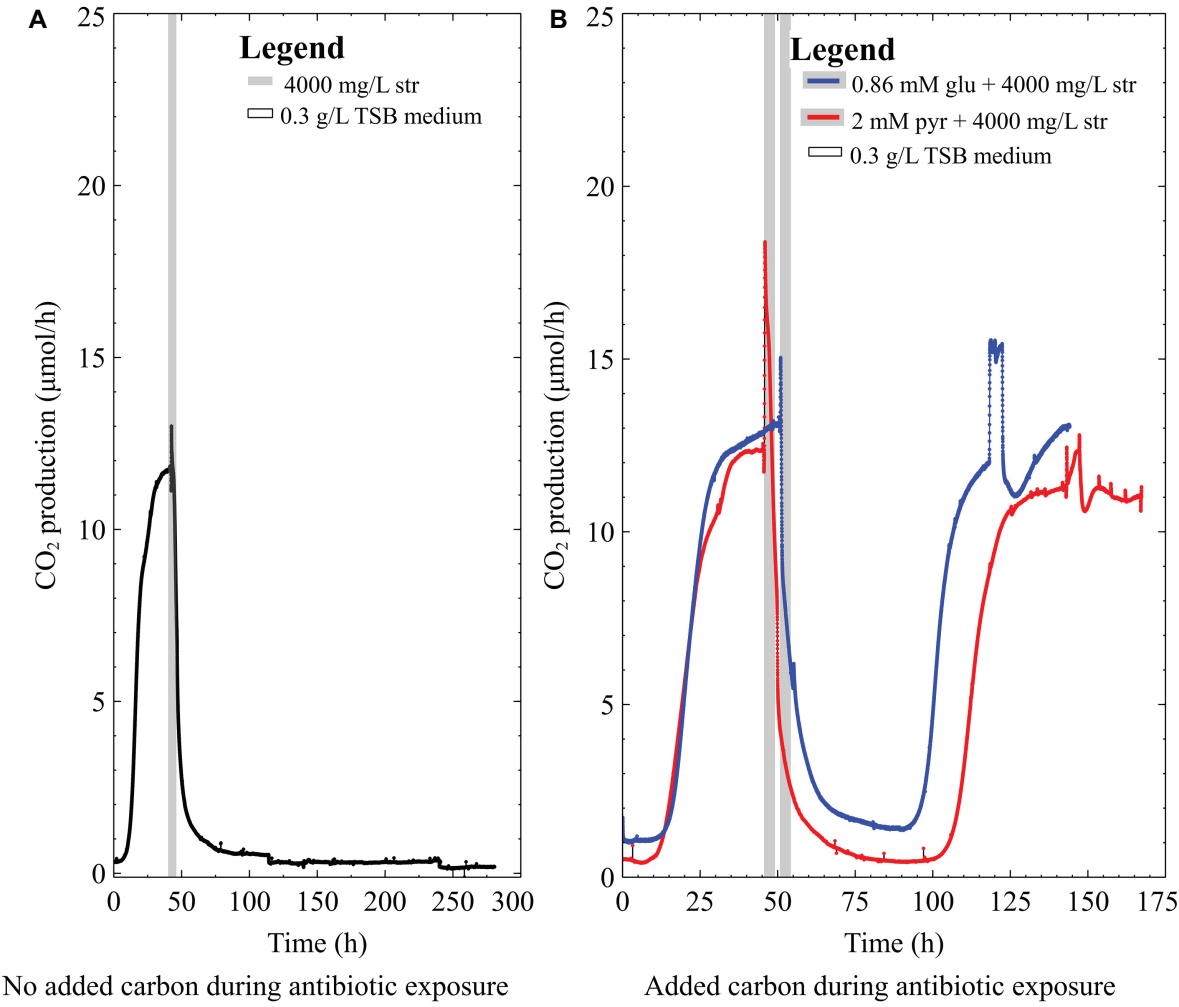
The CO_2_ production profile (μmol/h) of early steady-state multispecies biofilms grown in 0.3 g/L TSB medium exposed to **(A)** 4,000 mg/L streptomycin for 4 h, **(B)** 4,000 mg/L streptomycin for 4 h with either 2 mM pyruvate (pyr) or 0.86 mM glucose (glu) added to the antibiotic medium.

The mixed-species culture in this study contained both *P. aeruginosa* and *S. maltophilia*. This combination is relevant as *Stenotrophomonas* has frequently been co-isolated with *Pseudomonas* from infections and has a different metabolic route for glycolysis compared to *Pseudomonas* (Kanehisa and Goto, 2000; Tseng et al., 2009; Kanehisa et al., 2016). A 2-day-old mixed-species biofilm (Figure 4A) did not survive 4,000 mg/L streptomycin exposure in 0.3 g/L TSB medium (the main carbon source in TSB medium is glucose). However, when the mixed-species biofilms were exposed to antibiotics in medium supplemented with glucose or pyruvate they were able to survive (Figure 4B). Thus far, it has been demonstrated that nutritional status is important for the survival of both pure culture and mixed-species biofilms. Since the mere presence of *S. maltophilia* is not enough to confer resistance in the mixed-species biofilm, it was of interest to correlate the antibiotic data and the species composition of the biofilm *via* metagenomic analysis of the mixed-species biofilm.

**Figure 5:**
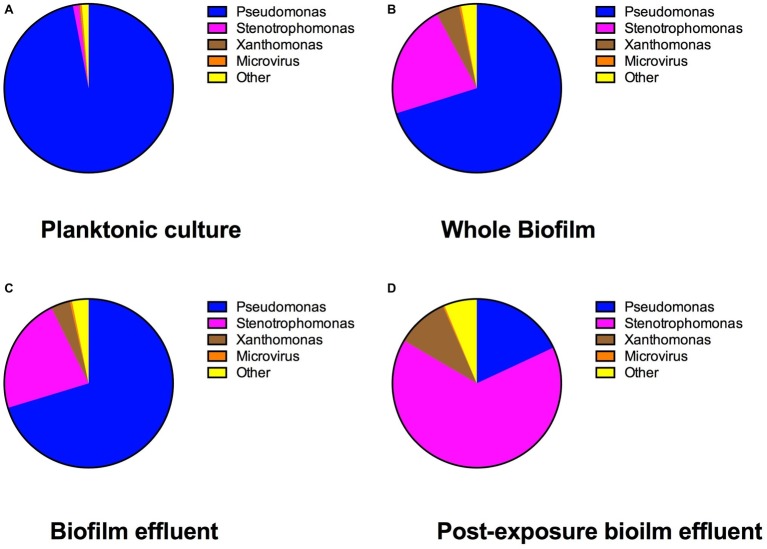
The percent genus distribution of bacterial 16S DNA present in the multispecies **(A)** freezer stock, **(B)** biofilm, **(C)** pre-streptomycin exposure effluent, and **(D)** post-streptomycin exposure effluent. This figure demonstrates how the multispecies community shifted from planktonic state to biofilm and following a streptomycin exposure in 0.3 g/L TSB medium.

**Figure 6:**
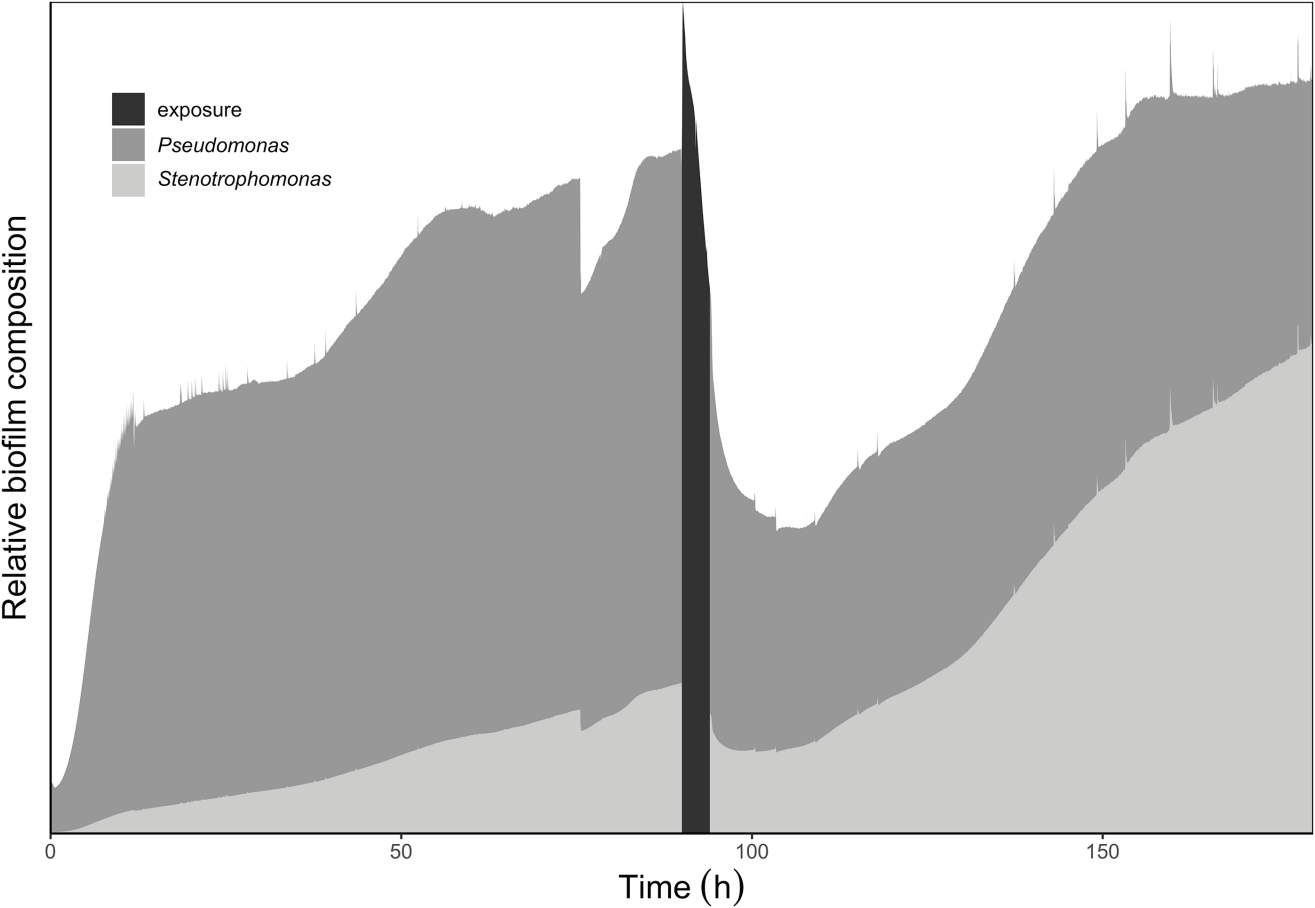
Relative abundance of *Pseudomonas* to *Stenotrophomonas* within the multispecies biofilm at various time points in biofilm growth. The data used for the schematic representation were genus distributions annotated from the metagenomes submitted to mg-RAST. The metagenomic samples taken had percentage (%) bacterial compositions (*Pseudomonas*: *Stenotrophomonas*) as follows: initial inoculum 97:1; steady-state biofilm and pre-exposure biofilm effluent: 70:20; post-exposure effluent 18:65. The schematic shows an increase in *Stenotrophomonas* in the population following inoculation and biofilm development. Following the antibiotic exposure the relative abundance of *Stenotrophomonas* is seen to shift again; reflecting the effluent sample taken following recovery after the antibiotic exposure.

The results of the bacterial identities at the genus level from mg-RAST are listed in Figure 5. The species abundance is derived from the number of annotations made at the species level from all the annotation source databases used by mg-RAST (Meyer et al., 2008). *P. aeruginosa* strains have four copies of 16S rRNA (Bodilis et al., 2012), and *S. maltophilia* has five to six copies (Zhu et al., 2012), which are comparable and why we are comfortable describing the species composition as is seen in Figure 5. The most common *Pseudomonas* strain annotated in each of the cultures was *P. aeruginosa* (PAO1) and the most common *Stenotrophomonas* strain annotated was *S. maltophilia* (R551-3). The whole-biofilm data and the pre-exposure effluent data show that *P. aeruginosa* accounts for ~70% of the species annotated in the biofilm metagenome and is the dominant organism before antibiotic exposure. It is important to note that biofilm composition will change with age and exposure to different nutrient regimes and stresses. The schematic shown in Figure 6 depicts the shift in relative species abundance over time. The schematic represents continuous CO_2_ production data (as a measure of total biomass, Bester et al., in preparation) overlaid with relative community ratios interpolated from discreet data points. As shown in Figure 5, a planktonic inoculum comprising of 97% *Pseudomonas* and 1.1% *Stenotrophomonas* resulted in a biofilm composition of 70.3% *Pseudomonas* and 22.4% *Stenotrophomonas* on day 3—indicating that the relative abundance of community members in the planktonic inoculum will not necessarily be maintained by the community composition of the biofilm it engendered. Interestingly we recorded a composition of 70.2% *Pseudomonas* and 21.9% *Stenotrophomonas* in the effluent before treatment; closely mirroring the ratio obtained for the whole-biofilm community (Figures 5B,C) suggesting that effluent composition approximates the biofilm composition. Following the antibiotic exposure and subsequent metabolic recovery back to steady state levels, the biofilm effluent shifted from a *Pseudomonas* dominated community to a *Stenotrophomonas* dominated community with a 65.5% *Stenotrophomonas* and 18.1% *Pseudomonas* distribution (Figure 5D).

## Discussion

This work highlights the importance of considering the nutritional status when interpreting biofilm survival in response to stressors. The results further supported the hypothesis that bacterial antibiotic susceptibility will differ in pure-culture according to the availability of readily degradable carbon compounds. Although not flowing directly from our hypothesis and predictions, we observed that survival of one community member in a biofilm favored by nutritional conditions can aid in the survival of community members that were vulnerable as a pure-culture under the same conditions.

Previous studies have shown that nutrient status during antibiotic exposure affects survival, where certain metabolites can either enhance or inhibit antibiotic susceptibility (Allison et al., 2011; Barraud et al., 2013; Jackson et al., 2015). In this work, early steady state mixed-species biofilms (~70% *P. aeruginosa*) and pure-culture PAO1 biofilms did not recover from high dose streptomycin exposures in TSB medium unless carbon was added to the antibiotic medium (Figures 2A, 3A). In the mixed-species culture, either pyruvate or glucose addition to the antibiotic medium aided in biofilm survival (Figure 4B), whereas in pure-culture *P. aeruginosa* biofilms only pyruvate (not glucose) addition aided in survival post-antibiotic exposure (Figures 3A,B). By contrast, the same TSB medium provided an environment suitable for pure-culture *S. maltophilia* biofilm survival, without the need for added carbon (Figure 2B). An increase in CO_2_ as observed in Figure 2B could result from changes in pH or increases in metabolism. It is not believed that a drop in pH is the cause of the increased metabolism, as biofilm exposures to pH as low as 5 had been performed without the large changes in CO_2_ output shown in Figure 2B (unpublished data). Therefore, it is believe that the rapid increase in CO_2_ output upon antibiotic exposure might result from a number of cellular processes that affect metabolism (for example efflux pumps, stress responses, or other homeostatic processes). In particular, *S. maltophilia* is inherently equipped with energy requiring efflux pumps and aminoglycoside modifying enzymes capable of effluxing streptomycin (Crossman et al., 2008), and these may account for the rapid metabolic increase seen in response to the high-dose streptomycin exposure in Figure 2B. In the mixed-species culture, it appears plausible that glucose addition aided the *Stenotrophomonas*, and the added pyruvate was aiding *Pseudomonas*, as *Pseudomonas* did not derive benefit from glucose addition in pure culture. This demonstrates the importance of ecological interactions in addition to nutritional status, as each species metabolize carbohydrates differently; a factor should be considered in the evaluation of antibiotics as it became increasingly evident that mixed-species infections are not the exception.

Although both *S. maltophilia* and *P. aeruginosa* can metabolize glucose, *P. aeruginosa* has been shown to preferentially metabolize amino acids and organic acids to glucose, when these carbon sources are all present in the growth medium (Ng and Dawes, 1967; Mukkada et al., 1973; Collier et al., 1996). Thus, in TSB medium, glucose may not be the primary carbon source utilized by *P. aeruginosa* during antibiotic exposure. Regarding glycolysis, *P. aeruginosa* metabolizes glucose *via* the Entner-Doudoroff pathway, which produces one less ATP compared to the Embden-Meyerhof-Parnas (EMP) pathway used by *S. maltophilia* (Kanehisa and Goto, 2000b; Stettner and Segrè, 2013; Kanehisa et al., 2016). However, further experimental evidence would be required to confirm that higher ATP production afforded such metabolic advantage that leads to the survival of *Stenotrophomonas* with glucose addition. Apart from being a carbon source and electron donor, carbon addition might also affect other environmental factors such as pH and osmolarity. However, since we observed survival of mixed-species biofilms under all conditions of carbon addition, we argue here that the added carbon did not directly interfere with the antibiotic action.

This study focused on the effect of nutritional status on antibiotic resistance in two common opportunistic pathogens (*P. aeruginosa* and *S. maltophilia*; Crossman et al., 2008). Previous work has shown that *S. maltophilia* colonization is not always associated with mortality and disease, but it can act as an indirect pathogen (Ryan et al., 2009). In this work, we demonstrated that the presence of *S. maltophilia* was aiding in whole biofilm survival under different environmental conditions, and more specifically in the survival of other pathogens.

A shift in community composition upon biofilm maturation that is different from the source of inoculum reveals how biofilm development can lead to competitive advantages for community members. The metagenomic community profile demonstrates that the established biofilm and the pre-exposure effluent population mirrored each other, but the effluent population composition shifted following an antibiotic exposure to a community dominated by *Stenotrophomonas.* The increase in *Stenotrophomonas* in the population following antibiotic exposure indicated that it had a competitive advantage compared to the *Pseudomonas* in the culture, either inherently or due to the environmental conditions at the time. Nevertheless, in the mixed-species setting, the mere presence of the *S. maltophilia* was not enough to guarantee survival for early steady state biofilms, while carbon addition could. Future work should attempt to uncover a threshold concentration of *Stenotrophomonas* needed for early steady state biofilm survival. The other species annotated in the cultures following metagenomic analysis (Figure 5) have probably played a minimal role in the ability to recover from the antibiotic exposures in this study. Others have pointed out that the DNA extraction kits and reagents used to prepare DNA samples for further analysis have widespread DNA contamination. Bacterial DNA commonly found contaminating extraction kits include several of the bacteria sequenced in this study such as *Pseudomonas*, *Stenotrophomonas*, *Burkholderia* (indicated under other), and *Xanthomonas* (Salter et al., 2014).

Therefore, it appears plausible that nutrient availability is a strong determinant of biofilm survival following antibiotic exposure, and it is worth considering the environment in which antibiotics are administered. Both pure-culture and mixed-species cultures containing *P. aeruginosa* were susceptible to the streptomycin exposures in TSB medium unless a nutritional boost was provided along with the antibiotic. As long as the addition of carbon could aid one of the community members, the biofilm survived. This work demonstrates the importance of ecological interactions in survival to stressors such as antibiotics, and thus, the need to emphasize the potential added complexity that mixed-species infections pose to health care. The observations made here may also have some relevance for nutritional conditions to apply when performing antibiotic minimum inhibitory concentrations.

## Data Availability Statement

The datasets generated for this study can be found in the https://www.mg-rast.org/: mgp12004, mgp11105, mgp12016.

## Author Contributions

All authors listed have made a substantial, direct and intellectual contribution to the work, and approved it for publication.

### Conflict of Interest

The authors declare that the research was conducted in the absence of any commercial or financial relationships that could be construed as a potential conflict of interest.

## References

[ref1] Allison K. R. Brynildsen M. P. Collins J. J. (2011). Metabolite-enabled eradication of bacterial persisters by aminoglycosides. Nature 473, 216–220. 10.1038/nature10069 21562562 PMC3145328

[ref2] Araoka H. Baba M. Yoneyama A. (2010). Risk factors for mortality among patients with *Stenotrophomonas maltophilia* bacteremia in Tokyo, Japan, 1996-2009. Eur. J. Clin. Microbiol. Infect. Dis. 29, 605–608. 10.1007/s10096-010-0882-6 20177726

[ref3] Barraud N. Buson A. Jarolimek W. Rice S. A. (2013). Mannitol enhances antibiotic sensitivity of persister bacteria in *Pseudomonas aeruginosa* biofilms. PLoS One 8:e84220. 10.1371/journal.pone.0084220 24349568 PMC3862834

[ref4] Beceiro A. Tomás M. Bou G. (2013). Antimicrobial resistance and virulence: a successful or deleterious association in the bacterial world? Clin. Microbiol. Rev. 26, 185–230. 10.1128/CMR.00059-12 23554414 PMC3623377

[ref5] Bester E. Kroukamp O. Wolfaardt G. M. Boonzaaier L. Liss S. N. (2010). Metabolic differentiation in biofilms as indicated by carbon dioxide production rates. Appl. Environ. Microbiol. 76, 1189–1197. 10.1128/AEM.01719-09 20023078 PMC2820951

[ref6] Bodilis J. Nsigue-Meilo S. Besaury L. Quillet L. (2012). Variable copy number, intra-genomic heterogeneities and lateral transfers of the 16S rRNA gene in Pseudomonas. PLoS One 7:e35647. 10.1371/journal.pone.0035647 22545126 PMC3335818

[ref7] Brooke J. S. (2012). Stenotrophomonas maltophilia: an emerging global opportunistic pathogen. Clin. Microbiol. Rev. 25, 2–41. 10.1128/CMR.00019-11 22232370 PMC3255966

[ref8] Brooun A. Liu S. Lewis K. I. M. (2000). A dose-response study of antibiotic resistance in *Pseudomonas aeruginosa* biofilms. Antimicrob. Agents Chemother. 44, 640–646. 10.1128/AAC.44.3.640-646.2000.Updated 10681331 PMC89739

[ref9] Bryers J. D. (2008). NIH public access. Artif. Cells Blood Substitutes Immobil. Biotechnol. Cells Blood Substit Immobi 100, 1–18. 10.1002/bit.21838.Medical

[ref10] Burmølle M. Webb J. S. Rao D. Hansen L. H. Sørensen S. J. Kjelleberg S. (2006). Enhanced biofilm formation and increased resistance to antimicrobial agents and bacterial invasion are caused by synergistic interactions in multispecies biofilms. Appl. Environ. Microbiol. 72, 3916–3923. 10.1128/AEM.03022-05 16751497 PMC1489630

[ref11] Collier D. N. Hager P. W. Phibbs P. V. J. (1996). Catabolite repression control in the pseudomonads.pdf. Res. Microbiol. 147, 551–561. 9084769 10.1016/0923-2508(96)84011-3

[ref12] Costerton J. W. Stewart P. S. Greenberg E. P. (1999). Bacterial biofilms: a common cause of persistent infections. Science 284, 1318–1322. Available at: http://www.jstor.org.ezproxy.lib.ryerson.ca/stable/2899085?pq-origsite=summon&seq=1#page_scan_tab_contents (Accessed July 7, 2015). 10334980 10.1126/science.284.5418.1318

[ref13] Crossman L. C. Gould V. C. Dow J. M. Vernikos G. S. Okazaki A. Sebaihia M. (2008). The complete genome, comparative and functional analysis of *Stenotrophomonas maltophilia* reveals an organism heavily shielded by drug resistance determinants. Genome Biol. 9:R74. 10.1186/gb-2008-9-4-r74 18419807 PMC2643945

[ref14] Damper P. D. Epstein W. (1981). Role of the membrane potential in bacterial resistance to aminoglycoside antibiotics. Antimicrob. Agents Chemother. 20, 803–808. 10.1128/AAC.20.6.803 6173015 PMC181802

[ref15] Davey M. E. O’Toole G. A. (2000). Microbial biofilms: from ecology to molecular genetics. Microbiol. Mol. Biol. Rev. 64, 847–867. Available at: http://www.pubmedcentral.nih.gov/articlerender.fcgi?artid=99016&tool=pmcentrez&rendertype=abstract (Accessed July 2, 2015). 11104821 10.1128/mmbr.64.4.847-867.2000 PMC99016

[ref16] de Carvalho C. C. C. R. (2018). Marine biofilms: a successful microbial strategy with economic implications. Front. Mar. Sci. 5:126. 10.3389/fmars.2018.00126

[ref17] Dowd S. E. Sun Y. Secor P. R. Rhoads D. D. Wolcott B. M. James G. A. (2008). Survey of bacterial diversity in chronic wounds using pyrosequencing, DGGE, and full ribosome shotgun sequencing. BMC Microbiol. 8:43. 10.1186/1471-2180-8-43 18325110 PMC2289825

[ref18] Eschbach M. Schreiber K. Trunk K. Buer J. Jahn D. Schobert M. (2004). Long-term anaerobic survival of the opportunistic pathogen *Pseudomonas aeruginosa* via pyruvate fermentation. J. Bacteriol. 186, 4596–4604. 10.1128/JB.186.14.4596-4604.2004 15231792 PMC438635

[ref19] Fraud S. Poole K. (2011). Oxidative stress induction of the MexXY multidrug efflux genes and promotion of aminoglycoside resistance development in *Pseudomonas aeruginosa*. Antimicrob. Agents Chemother. 55, 1068–1074. 10.1128/AAC.01495-10 21173187 PMC3067074

[ref20] Goss C. H. Otto K. Aitken M. L. Rubenfeld G. D. (2002). Detecting Stenotrophomonas maltophilia does not reduce survival of patients with cystic fibrosis. Am. J. Respir. Crit. Care Med. 166, 356–361. 10.1164/rccm.2109078 12153970

[ref21] Jackson L. Kroukamp O. Wolfaardt G. (2015). Effect of carbon on whole-biofilm metabolic response to high doses of streptomycin. Front. Microbiol. 6:953. 10.3389/fmicb.2015.00953 26441887 PMC4566048

[ref22] Kanehisa M. Goto S. (2000). KEGG: Kyoto encyclopedia of genes and genomes. Nucleic Acids Res. 28, 27–30. Available at: http://www.pubmedcentral.nih.gov/articlerender.fcgi?artid=102409&tool=pmcentrez&rendertype=abstract (Accessed July 10, 2014). 10592173 10.1093/nar/28.1.27 PMC102409

[ref23] Kanehisa M. Sato Y. Kawashima M. Furumichi M. Tanabe M. (2016). KEGG as a reference resource for gene and protein annotation. Nucleic Acids Res. 44, D457–D462. 10.1093/nar/gkv1070 26476454 PMC4702792

[ref24] Kataoka D. (2003). The indirect pathogenicity of *Stenotrophomonas maltophilia*. Int. J. Antimicrob. Agents 22, 601–606. Available at: http://resolver.scholarsportal.info/resolve/09248579/v22i0006/601_tiposm.xml (Accessed July 29, 2015). 14659658 10.1016/s0924-8579(03)00244-9

[ref25] Kroukamp O. Wolfaardt G. M. (2009). CO_2_ production as an indicator of biofilm metabolism. Appl. Environ. Microbiol. 75, 4391–4397. 10.1128/AEM.01567-08. 19346353 PMC2704800

[ref26] Lee H. H. Molla M. N. Cantor C. R. Collins J. J. (2010). Bacterial charity work leads to population-wide resistance. Nature 467, 82–85. 10.1038/nature09354 20811456 PMC2936489

[ref27] Lewis K. (2007). Persister cells, dormancy and infectious disease. Nat. Rev. Microbiol. 5, 48–56. 10.1038/nrmicro1557 17143318

[ref28] Mah T. F. C. O’Toole G. A. (2001). Mechanisms of biofilm resistance to antimicrobial agents. Trends Microbiol. 9, 34–39. 10.1016/S0966-842X(00)01913-2 11166241

[ref29] Marchand S. De Block J. De Jonghe V. Coorevits A. Heyndrickx M. Herman L. (2012). Biofilm formation in milk production and processing environments; influence on milk quality and safety. Compr. Rev. Food Sci. Food Saf. 11, 133–147. 10.1111/j.1541-4337.2011.00183.x

[ref30] Meyer F. Paarmann D. D’Souza M. Olson R. Glass E. M. Kubal M. (2008). The metagenomics RAST server—a public resource for the automatic phylogenetic and functional analysis of metagenomes. BMC Bioinform. 9:386. 10.1186/1471-2105-9-386 PMC2563014 18803844

[ref31] Mukkada A. J. Long G. L. Romano A. H. (1973). The uptake of 2-deoxy-D-glucose by *Pseudomonas aeruginosa* and its regulation. Biochem. J. 132, 155–162. 4199013 10.1042/bj1320155 PMC1177575

[ref32] Ng F. M. Dawes E. A. (1967). Regulation of enzymes of glucose metabolism by citrate in *Pseudomonas aeruginosa*. Biochem. J. 104:48 PMC1271291 4963525

[ref33] Nithya C. Begum M. F. Pandian S. K. (2010). Marine bacterial isolates inhibit biofilm formation and disrupt mature biofilms of *Pseudomonas aeruginosa* PAO1. Appl. Microbiol. Biotechnol. 88, 341–358. 10.1007/s00253-010-2777-y 20665017

[ref46] O’Toole G. Kaplan H. B. Kolter R. (2000). Biofilm formation as microbial development. Annu. Rev. Microbiol. 54, 49–79. 11018124 10.1146/annurev.micro.54.1.49

[ref34] Paerl H. W. Pinckney J. L. (1996). A mini-review of microbial consortia: Their roles in Aquatic production and biogeochemical cycling. Microb. Ecol. 31, 225–247. 10.1007/BF00171569 8661534

[ref35] Rojo F. (2010). Carbon catabolite repression in pseudomonas: optimizing metabolic versatility and interactions with the environment. FEMS Microbiol. Rev. 34, 658–684. 10.1111/j.1574-6976.2010.00218.x. 20412307

[ref36] Ronan E. Edjiu N. Kroukamp O. Wolfaardt G. Karshafian R. (2016). USMB-induced synergistic enhancement of aminoglycoside antibiotics in biofilms. Ultrasonics 69, 182–190. 10.1016/j.ultras.2016.03.017. 27111871

[ref37] Ronan E. Yeung C. W. Hausner M. Wolfaardt G. M. (2013). Interspecies interaction extends bacterial survival at solid–air interfaces. Biofouling 29, 1087–1096. 10.1080/08927014.2013.829820. 24041248

[ref38] Ryan R. P. Monchy S. Cardinale M. Taghavi S. Crossman L. Avison M. B. (2009). The versatility and adaptation of bacteria from the genus Stenotrophomonas. Nat. Rev. Microbiol. 7, 514–525. 10.1038/nrmicro2163 19528958

[ref39] Salter S. J. Cox M. J. Turek E. M. Calus S. T. Cookson W. O. Moffatt M. F. (2014). Reagent and laboratory contamination can critically impact sequence-based microbiome analyses. BMC Biol. 12:87. 10.1186/s12915-014-0087-z 25387460 PMC4228153

[ref40] Schiessl K. T. Hu F. Jo J. Nazia S. Z. Wang B. Price-Whelan A. (2019). Phenazine production promotes antibiotic tolerance and metabolic heterogeneity in *Pseudomonas aeruginosa* biofilms. Nat. Commun. 10:762. 10.1038/s41467-019-08733-w 30770834 PMC6377615

[ref41] Schreiber K. Boes N. Eschbach M. Jaensch L. Wehland J. Bjarnsholt T. (2006). Anaerobic survival of *Pseudomonas aeruginosa* by pyruvate fermentation requires an Usp-type stress protein. J. Bacteriol. 188, 659–668. 10.1128/JB.188.2.659-668.2006 16385055 PMC1347276

[ref42] Stettner A. I. Segrè D. (2013). The cost of efficiency in energy metabolism. Proc. Natl. Acad. Sci. USA 110, 9629–9630. 10.1073/pnas.1307485110 23729810 PMC3683743

[ref43] Stewart P. S. (2002). Mechanisms of antibiotic resistance in bacterial biofilms. Int. J. Med. Microbiol. 292, 107–113. 10.1078/1438-4221-00196 12195733

[ref44] Stover C. Pham X. Erwin A. Mizoguchi S. (2000). Complete genome sequence of *Pseudomonas aeruginosa* PAO1, an opportunistic pathogen. Nature 406, 959–964. Available at: http://www.nature.com/nature/journal/v406/n6799/abs/406959a0.html 10984043 10.1038/35023079

[ref45] Tan C. H. Lee K. W. K. Burmølle M. Kjelleberg S. Rice S. A. (2017). All together now: experimental multispecies biofilm model systems. Environ. Microbiol. 19, 42–53. 10.1111/1462-2920.13594 27878947

[ref47] Tseng C.-C. Fang W.-F. Huang K.-T. Chang P.-W. Tu M.-L. Shiang Y.-P. (2009). Risk factors for mortality in patients with nosocomial *Stenotrophomonas maltophilia* pneumonia. Infect. Control Hosp. Epidemiol. 30, 1193–1202. 10.1086/648455 19852664

[ref48] Xiong Y. Q. Caillon J. Drugeon H. Potel G. Baron D. (1996). Influence of pH on adaptive resistance of *Pseudomonas aeruginosa* to aminoglycosides and their postantibiotic effects. Antimicrob. Agents Chemother. 40, 35–39. 8787875 10.1128/aac.40.1.35 PMC163052

[ref49] Zhang L. Li X. Z. Poole K. (2000). Multiple antibiotic resistance in *Stenotrophomonas maltophilia*: involvement of a multidrug efflux system. Antimicrob. Agents Chemother. 44, 287–293. 10.1128/aac.44.2.287-293.2000 10639352 PMC89673

[ref50] Zhu B. Liu H. Tian W.-X. Fan X.-Y. Li B. Zhou X.-P. (2012). Genome sequence of *Stenotrophomonas maltophilia* RR-10, isolated as an endophyte from rice root. J. Bacteriol. 194, 1280–1281. 10.1128/JB.06702-11 22328769 PMC3294802

